# Remarks on Professional Morality

**Published:** 1839-08

**Authors:** Solyman Brown


THE AMERICAN JOURNAL

OF

DENTAL SCIENCE.

Vol. I. — No. II.

### REMARKS ON PROFESSIONAL MORALITY.

[BY SOLYMAN BROWN, A. M.]

To men of high-toned sensibility, animated by that "*esprit du corps*" from which much of the energy of human action, and still more of the enthusiasm of human feeling, result, it can be matter of no trifling moment that the profession to which they belong—with which they stand associated in the public mind—and from which they derive a livelihood, if not a fortune, should be at once respectable and respected, and so far removed from any general and well founded reproach as to rank among the varied avocations of society according to its positive utility.

The profession in which the practical dentist is engaged, is one so generally in demand—so endeared to community by the relief which it brings to human suffering—and requiring so much skill and talent to elevate it to its possible perfectibility, that nothing but the character of its professors has restricted its influence and usefulness in ages past, or shall in times to come, mar its reputation, or circumscribe its emolument.

Dentistry in its improved forms, is one of those new sciences by which the present age is distinguished—and although we have reason to suppose it had attained to some celebrity in the Augustan age of Rome, we may well ascribe to our own times, and even to the present century, the highest advancements in our art, known to authentic history.

No sooner is it discovered that any art or science must of necessity be incorporated in the texture of society, than it becomes demonstrable that its successful pursuit will be a permanent passport to fame and fortune; and when this was ascertained to be the condition of our professional science, it was not to be doubted that numerous adventurers would flock to its temple. This reasonable suspicion has been amply verified in the fact, that there are now almost a hundred individuals calling themselves Dentists, in the city of New-York alone. But such a fact would only betoken the prosperity and popularity of our art, and therefore become a subject of congratulation among its honorable professors, were it not one of the results of this great accession of numbers, that much professional incapacity and great moral delinquency of character, become thus connected with our calling.

It is quite true that all other professions of equally recent institution, are liable to the same inconvenience ; and even those the longest established, however honorable and useful, do not entirely escape it.

The science of Medicine and Surgery, so much akin to our own, has its quacks and mountebanks, as the newspapers of the day, and the dupes of their prescriptions too amply testify. Nor is the most sacred of all human functions wholly preserved from the charlatanism of voluntary ignorance and graceless presumption. There are quack doctors in the desk as truly, although we hope not as frequently, as in the laboratory, and the effects of their nostrums cannot be less disastrous.

Since, then, both that profession which consults the health of the body of man, and that which should cultivate the welfare of his soul, are alike disgraced by charlatans, let us not suffer ourselves to be wholly inconsolable, because quackery, with its detestable allies, Ignorance, Recklesness and Ostentation, has polluted the threshold of the temple in which our favorite science sits permanently enthroned. Let us rather submit as gracefully as possible to the necessity of our condition, finding some consolation in the fact that all human avocations are subject to equal disadvantage, and allowing ourselves to be especially gratified in reflecting that time will, in some measure, correct the evil. The process of fermentation must, for a while, elevate froth and filth to the surface; but the liquor beneath becomes purer and better as it stands ripening upon its lees !

It is our duty, as good citizens, to labor *collectively*, as well as individually, for the elevation of our profession in character and in usefulness.

It will be my design to show, if indeed it be not manifest already, that the usefulness of our art can never become general, much less universal, until the standard of *professional morals* shall be greatly elevated, and this happy change become known to community at large. I should willingly excuse myself from the painful duty of calling the attention of my brethren too particularly to those deep stains upon the character of our profession, by which the confidence and respect of the public have been so sadly shattered, if I were not fully apprized of the willingness of all good men, to look at the evil in its utmost enormity, and their determination to adopt a remedy co-extensive with the disease. We have all felt keenly the deepest chagrin and indignation at being suspected in our veracity, merely because we are connected with a class of individuals in whose moral character and professional habits, mendacity is a predominant ingredient. How mortifying to a generous mind to be classed in even the remotest alliance with men whose tongues may well be festered by the falsehoods which they utter !

I must entreat my readers not to charge me with undue or unwarrantable warmth on this occasion. With such a theme the language of indignation can scarcely become intemperate. For my own part, I should deem myself justified if such a thing were possible, in selecting on this topic, from the vocabularies of human language, such burning words as should scorch the lips that uttered, and the ears that imbibed them.

To those among us who have been long in the profession, or who have been otherwise for many years conversant with the history of Dentistry throughout these United States, the many dishonest arts by which the public have been disgusted and abused, are already known. Every practitioner becomes acquainted with histories of baseness, related by his patients, which well nigh cause the blood to curdle in his veins; and although some allowance is always to be made for exaggeration and misrepresentation on the part of the accusers, there can be little doubt of the general truth and propriety of their complaints.

Our memories are still fresh with the recollection of those splendid hum-bugs, the Messrs. Crawcour, who made so successful a requisition of some thousands of pounds of their own sterling currency, upon the purses of our fellow citizens, for having imported from England a more direct and explicit method of applying mercury to the destruction of the teeth, than that so successfully practised by our native quacks through the medium of the circulating system. And I am pained to hear that this execrable material is still used by some unprincipled men in this country, and by one who has recently left this country, and is now practising in one of the West India Islands, who has the dishonesty to use it under the disguised name of "*paste*," to which he has affixed a fine and distinguishing appellation, and the effrontery to say that it is used and approved by the first dentists among us. Such high-handed impudence and villainy, cannot long escape the punishment it so richly deserves.

And here let it not be said that the folly of our fellow citizens was but too manifest in this "*coup de grace*" of these gasconading empyricks—for it should never be forgotten that the simplicity of the dupe is no palliation of the guilt of the deceiver. But these graceless gallows-hunters have been scouted from our shores, and we are left to grapple only with the minor spirits of this disgraceful "*coterie.*"

Let us, then, turn for a moment, to the habits and practices of some of our countrymen, who desecrate the name of Dentistry by their daily conduct.

What, think you, of a man who will not only advertize himself as the manufacturer of incorruptible mineral teeth, but seek and fraudulently obtain a premium at the Annual Fair for such manufacture, notwithstanding he never made so much as one such tooth in his life? Can you esteem such a man as a whit more honorable than he who publishes to the world the falsehood that he belongs to a Dentists' Society which never had existence?

What think you of him who, in this age of science and mutual interchange of knowledge, can gravely assure his patients that he is the original discoverer and only manufacturer of Creosote, the well known and abominable result of the distillation of tar?

What shall we say of the astonishing chemico-dental discoverer, who has found among the products of his laboratory, a demulcent elixir, by which, in a moment he can so soften the texture of the soundest tooth as to be able with a pair of pliers, to bend it in any desired direction, and to any required extent? Can we consider this successful alchemist as greatly surpassed by that other son of genius, who adapts artificial teeth to all the useful purposes for which these organs are designed, on the truly philosophical principles of capillary attraction and atmospheric pressure!

What shall we say of a Dentist who will extort a hundred and sixty dollars from an unprotected female from a remote section of our country for work executed at a single sitting, and in the compass of three hours, merely because she was too polite to ask his prices, and higgle for a penny, before the work was commenced?

And finally—how shall we classify, if not among quacks and impostors, the man who can wish to persuade the credulous multitude, that he can extract the teeth of men, horses, and wild boars, without pain or effort, by the simple use of a rusty jack-knife or a two-penny whistle?

But I will not weary you with examples of odious imposition, by which the annals of Dentistry are daily polluted. Let us rather ascend from particular instances to general principles, and inquire for that code of professional morals which should regulate our intercourse with our fellow men.

I will not allow myself to doubt for a moment, that each of my readers will admit the validity and binding force of the only moral rule which can be considered as the universal and unexceptionable law of genuine charity:—namely, "That we should do to others precisely as we would desire, and could reasonably require others to do to us in exactly corresponding circumstances." I say, this law in all its bearings,—"gradations just, and nice dependencies,"—is the divine law of social benevolence, and there can be no immaginable case in which it will not properly apply to the conduct of every rational individual.

Our daily calling which brings us into constant intercourse with our fellow men, is the proper sphere in which this law of love should operate; and he who imagines that charity and high toned christian morals are very becoming in our religious connexions, and in our domestic relations, but have little to do with our business transactions—they being of a worldly nature—has, in my opinion, lost the immortal substance of true religion by grasping at its pale and meagre shadow! In truth, every bargain which we negociate, and every charge which we make in the ordinary current of our daily business, should be habitually subjected to the mental inquisition,—"am I doing to others as I should reasonably desire others to do to me?" There is no safe subterfuge, no possible escape from this conclusion. The subtle sophistry of self-love cannot countervail; the soporific elixir of moral apathy cannot neutralize it. It stands recorded in words of light, and they cannot be too brightly written on every heart.

This then is the law—and in spite of all voluminous and learned commentaries by which its spirit may be assailed, it will never be dissipated from the good man's mind; but will remain the rule of duty, and therefore the law of happiness forever.

Human laws may indeed be less rigid, because they cannot reach the heart; but human laws—mere legislative enactments, regarding only the outward conduct, will never be the standard of morals with any individual who has a loftier ambition, and a nobler aim, than merely to escape the present punishment of his crimes.

It would be very easy for any one intimately acquainted with the details of dental practice, to apply this rule of action to numerous examples; and there is no profession in civilized society, those of the physician and clergyman perhaps excepted, in which the operations of this rule come home more directly to the interests of mankind, than that in which we are engaged. Ours is not a profession that ministers merely to the folly and vanity of mankind; that aims at exalting one man above another in splendor of equipage and tinsel of embellishment; that labors to create artificial distinctions in society, in order that the great may oppress the small; that contrives to concentrate all the floating wealth of the community into the hands of a few rapacious individuals who shall be thereby entitled to riot on the hard earned products of honest labour: the province of our art is more generous and noble; it wipes the tear from the cheek of beauty; extracts the pang from the mortal frame writhing with agony; and, if it gives not bread to the hungry, it enables the hungry to eat, and the dyspeptic to appropriate that bread which Providence has given them. Ours is a profession, which, if properly conducted, might be brought home to the happy acquaintance of every family in Christendom. The rich and the poor might all be induced to esteem it as equally essential to the well being of society, as that of their well beloved physician, or of the surgeon who wounds but to cure.

It may then be safely averred, that the above law of charity requires the Dentist often to exercise his professional skill, and devote a portion of his time to cases from which he receives little or no pecuniary emolument. The physician and surgeon often do this; and why should the dentist be exempted from the same duty?

There are instances in the professional experience of every dental practitioner, in which it would be barbarous to send away a patient without affording relief; and worse than oppressive to receive any pecuniary reward for his services. Although money is the god of this world, yet there are many opportunities presented to men of all professions, of worshiping the God of universal nature in the form most useful and acceptable, by doing good to others without reward.

This species of charity, however, is confined in most instances, to the relief of actual suffering, and applies not to that portion of a dentist's practice which may be supposed to respect mainly personal appearance. Of this distinction, every practitioner must be his own judge, since no other person can determine for him the exact point of duty.

Will there be thought to be any thing strange or peculiar in the circumstance, that the conscientious practitioner of our art should find frequent opportunities of performing acts of legitimate benevolence in the course of his professional practice? Is it not the purpose of Divine Providence that every condition of human life should present such opportunities of exercising the benevolent affections; and of thus imitating the celestial Benefactor who has freely given the riches of the universe to the creatures of his power?

Again. The honorable dental practitioner will adopt such a tariff of charges as will not be extortionate, or disproportionate to the profits of the other professions on the one hand; nor degrade his calling to the level of a vulgar art, on the other.

This proposition will be readily perceived to result from the general law of professional morality, to which we have already alluded.

It may perhaps be said that where a profession is untramelled by legal monopoly, and thus open to fair competition, there can be no extortion in demanding the most exorbitant prices, provided the parties be apprized beforehand of the expense to be incurred. The truth of this position I am not anxious to impugn; nor do I by any means object to the respectable charges made by those honorable gentlemen of our profession, who have raised themselves to an enviable distinction as well in fame as fortune, by their talents and their virtues.

But these concessions do not require me to abandon my proposition, that the honorable practitioner will neither be oppressively high nor meanly low in his professional charges. Either of these extremes would bring detriment to society, and therefore would be contrary to genuine charity; because, very few individuals compared with the mass of community, could afford to pay exorbitant pri es, and still fewer would be benefitted by our profession, if it were exercised by those persons only who would labor for a mean compensation.

I shall best express my meaning on the subject of extortionate charges, by alleging an instance which occurred in the city of New-York within a few months past, and which was related to me by the highly respectable individual who was the intended victim of outrageous extortion.

A lady from a Southern city, induced by the flaming and flagrantly mendacious advertisement of one of the Dentists of N. York, called upon him on her arrival in town to inquire the price of a double set of artificial teeth. After learning the object of her visit, and ascertaining that she was a stranger in the city, from one of the cotton planting states, he assured her that his regular price for such a set of teeth was *fifteen hundred dollars!* I hardly need remark that such a demand, whether made before the work was done or after it was completed, was equally wicked and extortionate, and can rank in no better society than Fraud, Impudence and Quackery.

The enormity of the crime of extortion in our profession, in those instances in which the prices are unknown until the work has been completed, is enhanced by the fact, that persons of sensibility, and especially delicate females, are deterred from that redress which an appeal to the laws might give, by their aversion to exposure on such a subject.—The man, therefore, if indeed I do not vilify the race by calling him a man, who could stoop to acquire his livelihood—and much more to amass a fortune by such ill-got gain—would have been a bandit among the Appenines, or a pirate on the ocean. He would deem himself honored by being the hangman at Tyburn for a shilling a day; and would have gloried in distributing the poison *gratis* in the holds of the British prison ships during the war of our revolution. To such a man, the voice of truth indignantly exclaims, "Oh, full of all subtlety and all mischief, thou enemy of all righteousness, and thou child of the devil," when wilt thou cease to be a Quack and a Villain!

But I must suppress the sentiments of detestation so amply excited by those vagabonds, who bring indellible disgrace upon our calling, and proceed to remark, that the opposite evil of regulating the tariff of charges on so mean a scale as that competent, respectable and well bred men, could not afford to follow the profession, is sufficiently manifest; and we have all learned in our intercourse with our patrons, that the public are perfectly willing to reward the skilful dental surgeon as liberally as they do their physician, their lawyer, or their clergyman; as some of the members of our profession have learned by happy experience.

Once more. A just regard to professional honor, demands of the dental surgeon the most scrupulous punctiliousness and care in performing every operation in such a manner as he would himself wish and expect in a change of circumstances in which he were the patient and his patient the operator; or, were his wife or his child the subject of his treatment. Inordinate self-love is indeed little prone to institute such comparisons, but genuine morality requires it. When an individual with respectful confidence surrenders himself to the treatment of the Dental operator, he commits to him an important trust with an implied, if not an expressed stipulation, that it shall not be violated. No matter whether the patient demand verbally or not that the case shall be treated with the operator's best skill and judgment; no matter whether the subject be rich or poor, distinguished or obscure; the fact that he is a fellow creature, as fond as ourselves of health and happiness, entitles him to the full benefit of the moral precept we have learned from the Divine Law-giver. The love of gain should never beguile us so far from duty as to induce us to perform an operation when it is not required, merely because a patient requests it. Individuals often request an operation in their own case, which it would be mal-practice to perform; and even parents not unfrequently solicit the extraction of the first teeth of their children at a period when the safety of the second set would be endangered. In all such cases the practitioner's sense of propriety, and not the opinion of others, should be his guide.

The teeth are of so much importance to the well-being of the animal system, that all operations upon these organs should be performed not only with discretion but in good faith; and I can imagine no better rule of practice than to perform the duties of our profession in every instance as we would wish them performed were the case our own.

I proceed to remark, that a benevolent regard for the public welfare, forbids that any dentist should pretend to qualify students for general practice, by giving access for a few weeks only to his office, and a general certificate of skill and competence. The truth is, the profession of Dentistry is both an art and a science; an art that requires great mechanical skill, and a science demanding much mental cultivation. Such an art never can be acquired without long practice, especially by one who has not been bred at some kindred calling:—and such a science should claim years of research and general mental culture; particularly of those whose previous education has not been strictly if not technically prepa ratory.

Most other professions are protected either by statute or organized associations, from the encroachments of incompetence and imposture.—Ours is guarded by no civil enactments, and of consequence, the members should supply the defect either by their individual or collective efforts.

To effect so desirable an object, was one of the leading motives in the establishment of this JOURNAL.

The fact that a few individuals have acquired reputation and emolument by an extensive Dental Practice conducted with talent an integrity, has induced many adventurers in these days of experiment and specula tion, to rush hastily, and therefore unprepared into the lists of competition for public patronage; but there can be no reasonable doubt that to this state of things time and experience will apply a remedy. At any rate, it becomes the duty of every honorable practitioner to abstain from any acts that may augment the evil, how pleasing soever it might be to have either the reputation or the profits resulting from the office of instructer.

Again. Genuine professional morality requires of us to treat the good name of our fellow labourers in Dental Practice, with all the kindness and candor which we solicit for ourselves. We should neither feel nor act as the *rivals* of our brethren; but as *neighbours* in that sense of the term in which it was applied to the good Samaritan. For the exercise of this species of good will, there is constant occasion in our daily intercourse with patients and others; and this frequency of recurrence, in any charity, imparts to it superior importance.

But this rule does not require us to recommend or eulogize those men calling themselves dentists, who are in the known habit of abusing the confidence of others. To speak in exalted terms of such men, would be to confound virtue and vice; and, therefore, would be no charity; because it would be the means of beguiling the innocent to certain disappointment. There can be no virtue in assisting one man to impose upon another.

But of those individuals who are labouring with honest zeal, and employing creditable and useful talents in our art, for the honorable purpose of performing their social and domestic duties, I would always speak with the tenderest regard, and the most affectionate good will.

That narrow-minded jealousy which has so long and so notoriously disgraced the kindred profession of medicine and surgery, should be utterly repulsed from every good man's mind. If we see others even more successful than ourselves in the performance of extensive uses, as well as in the pursuit of fame and fortune, we should imitate the noble Roman who, when an unsuccessful candidate for the office of Decemvir, manfully replied to his wife, who informed him of his failure, "Well, my love, I am sincerely rejoiced that there are ten men in Rome better than myself."—Happy in his Elysian Fields is that generous spirit which felt on earth so nobly! And shall we, my brethren, who have been lighted in the path of truth by the Star of Bethlehem, suffer ourselves to be surpassed in high morality by Pagans?

Let us not forget, that, whether he reside in a Heathen or a Christian land, that man is but a sorry philanthropist, who finds not a portion of his happiness to consist in witnessing the prosperity of others.—

Nor let us ever cease to despise the wretch who would wantonly employ the authority of his opinion to degrade his professional competitors.—Such a man deserves a solitary cavern in the mountains of the moon.

*Finally*—It is the charitable duty of the honorable dental practitioner, to impart to others who are worthy, any valuable improvements and useful discoveries which he may make in his art. It is one of the distinctive traits of quackery to have—or pretend to have—professional *secrets*.—Knowledge in all those departments of science or of art, in which the public good is intimately involved, should be as free as the sunlight and as universal as the atmosphere.

Let us, then, once for all, settle it in our minds, that he who deals in nostrums of which the mystic ingredients are known only to himself; who boasts of performing the manipulations of his art with a skill of which he alone is possessed; who performs secret wonders known only to his fortunate and grateful patients; and who deals in mystery and concealment as though they were the distinguishing attributes of genius, is wholly unworthy of any of the liberal professions.

One of the fundamental principles of genuine science, is a community of truth; and I rejoice that those members of our profession who have had the most varied experience, and who enjoy the highest reputation, have been unreservedly liberal in this behalf. It is fondly hoped that the "AMERICAN JOURNAL OF DENTAL SCIENCE," will for years to come, afford to such men the means of doing good which is the true felicity, by giving popularity to knowledge which is the sister of virtue.

